# A new method called MiKneeSoTA to minimize knee soft-tissue artifacts in kinematic analysis

**DOI:** 10.1038/s41598-024-71409-z

**Published:** 2024-09-05

**Authors:** Ann-Kathrin Einfeldt, Leon Budde, Ariana Ortigas-Vásquez, Adrian Sauer, Michael Utz, Eike Jakubowitz

**Affiliations:** 1 Laboratory for Biomechanics and Biomaterials, Department of Orthopaedic Surgery, Hannover Medical School, Anna-von-Borries-Str. 1-7, 30625 Hannover, Germany; 2 Leibniz Universität Hannover, Institute of Mechatronic Systems, An der Universität 1, 30823 Garbsen, Germany; 3 Research and Development, Aesculap AG, Am Aesculap-Platz, 78532 Tuttlingen, Germany; 4 Department of Orthopaedic and Trauma Surgery, Musculoskeletal University Center Munich, Campus Grosshadern, Ludwig Maximilians University Munich, Marchioninistraße 15, 81377 Munich, Germany

**Keywords:** Musculoskeletal system, Biomedical engineering

## Abstract

The use of marker-based optical motion capture to estimate joint kinematics during gait is currently limited by errors associated with soft-tissue-induced motion artefacts (STIMA) and ambiguity in landmark palpation. This study therefore presents a novel protocol aiming to **Mi**nimize **Knee So**ft-**T**issue **A**rtefacts (MiKneeSoTA) and their effect on kinematic estimates. Relying on an augmented marker set and a new inverse kinematics approach, our method leverages frame-by-frame optimization to adjust best-fit cylinders that have been automatically generated based on the relative position of lower limb markers during an initial static trial. Tibiofemoral rotations and translations are then calculated along the anatomical joint axes based on the relative 3D motion of these cylinders. When compared against the conventional Helen-Hayes approach, in vivo assessment of fifteen healthy subjects revealed the MiKneeSoTA approach led to kinematic profiles with significantly lower standard deviations in joint rotations across trials, and even visibly reduced the presence of high frequency fluctuations presumably associated with e.g. soft-tissue vibration. In addition to agreeing with previously published bone pin and fluoroscopy datasets, our results illustrate MiKneeSoTA’s ability to abate the effect of STIMA induced by lateral knee ligaments. Our findings indicate that MiKneeSoTA is in fact a promising approach to mitigate knee joint STIMA and thus enable the previously unattainable accurate estimation of translational knee joint motion with an optoelectronic system.

## Introduction

A six-degrees-of-freedom (6DOF) joint, the tibiofemoral knee is the largest joint in the human body. A comprehensive understanding of the functional mechanisms of the knee joint is fundamental in a clinical context, especially in the development of efficient restoration strategies to treat injuries and disease^1^. Physiological kinematics of the tibiofemoral joint involve a complex interplay of rotational and translational motion, as well as a presumed dependency on the magnitude of knee flexion^2^. For example, past research has found evidence of both larger roll-back of the lateral femoral condyle with increasing flexion angles, as well as of a corresponding external rotation of the femur relative to the tibia^3^. The complex nature of tibiofemoral kinematics is further exacerbated by the presence of mechanical constraints imposed by soft tissue structures, whereby e.g. the anterior cruciate ligament (ACL) limits posterior femoral translation during flexion^4^. Clinical observations of injured or pathological patients clearly highlight how even subtle changes in joint physiology—such as cartilage abrasion due to osteoarthritis, or the stretching or tearing of ligaments due to injury—can easily have significant effects on the motion of the knee joint in multiple planes and along multiple axes^4–6^.

Extensive research with the overarching objective of empirically characterizing the unique kinematic patterns of the tibiofemoral joint has been performed in recent decades^1,7–10^. The method most frequently used to measure and describe human joint motions is optoelectronic marker-based motion capture^11,12^. This gait analysis approach involves attaching retroreflective markers to so-called anatomical landmarks (usually identified via simple palpation) and capturing their three-dimensional position over time using infrared cameras in a laboratory environment. Unfortunately, marker-based methods are highly susceptible to systematic errors often stemming from difficulties identifying non-discrete anatomical landmarks using unreliable manual palpation, as well the use of reflective markers placed over soft tissue layers of varying thickness to represent underlying skeletal features that can move relative to these markers due to soft tissue induced motion artefacts (STIMA)^13,14^. These errors affect not only the sagittal plane, but in fact usually also propagate into the other two secondary planes of motion^7^. As a result, different and sometimes even contradictory dynamic angle profiles for knee joint motions have been reported in the literature^12^.

Kadaba et al.^7^ showed that incorrect marker placement and therefore different knee axis definitions led to different joint angle profiles with either ab- or adduction during the swing phase of gait. In the transverse plane of motion, an offset where joint angle profiles start at different (internal or external) rotation values could be observed, even though the general joint angle pattern stayed the same. A separate study quantified STIMA by comparing data from skin markers versus intracortical bone pins across three subjects^15^. Results showed that errors in the frontal and transverse planes were considerably large relative to the magnitude of the motions, while errors affecting motion in the sagittal plane were significantly smaller. Further studies analyzing knee joint kinematics during activities characterized by large increases in flexion angle show high-frequency oscillations in the frontal plane. These fluctuations in ab/adduction observed during, for example, single-leg landings^12^ or the swing phase of gait^6,16^, could plausibly be indicative of STIMA effects.

Given the known limitations and (over-)simplification of most marker models, it is not uncommon for researchers to argue that kinematic measurements are only reliable if obtained along the primary plane of motion, such as the sagittal plane (and therefore flexion/extension) in case of the knee joint for most gait activities^14,15,17,18^. Existing marker models are arguably not precise enough to accurately characterize motion in the secondary planes due to STIMA. Looking at translational joint kinematics beyond these secondary rotations based on marker-based systems is generally considered futile, limiting the scope of optoelectronic gait analysis as a valuable method for capturing knee kinematics.

Although alternative methods of measuring knee joint motion patterns exist^1,8,19,20^, these are analogously subject to limitations. The use of dynamic MRI images, for example, is restricted for gait analysis due to spatial limitations stemming from the large size of the equipment. Cortical bone pins, on the other hand, are overly invasive, while dynamic fluoroscopy inevitably exposes subjects to potentially hazardous radiation, and thus their implementations are largely avoided due to ethical considerations.

To fulfill the need for a non-invasive motion capture method capable of accurately measuring knee joint kinematics, attempts have been made in the past to optimize existing marker models such that rotations in the frontal and transverse planes can be captured more precisely, and possibly even estimate the associated translational joint motions^11,13,21,22^. Reflective markers have, for example, been grouped into rigid clusters, which were then attached to each of the two leg segments. Using the local coordinate systems defined by the marker clusters, the relative displacements of femur and tibia were calculated for an activity^22^. Although this and other methods have indeed managed to provide an improved resolution of knee joint motion measurements compared to previous classic marker models, they not only showed differences between each other, but also notably deviated from results obtained by bone pin and fluoroscopy measurements^1,12^.

To close this gap, we address the need for a more accurate approach for the calculation of 6DOF tibiofemoral joint kinematics based on marker-based optical motion capture, by describing the theoretical foundation and detailed implementation of an alternative marker set and computational method. The proposed new marker set leverages an overdetermination of the two leg segments (thigh and shank) combined with a post-processing optimisation approach to ultimately **Mi**nimize **Knee So**ft-**T**issue **A**rtefacts (MiKneeSoTA) induced by motion, and additionally enable the evaluation of translational knee motions using conventional optoelectronic motion capture. The resulting kinematics of the presented MiKneeSoTA method are compared to the classical Helen-Hayes approach (also known as Plug in Gait model), as well as to established experimental knee motion analyses^1,8^ during normal level walking.

## Materials and methods

**Fig. 1:**
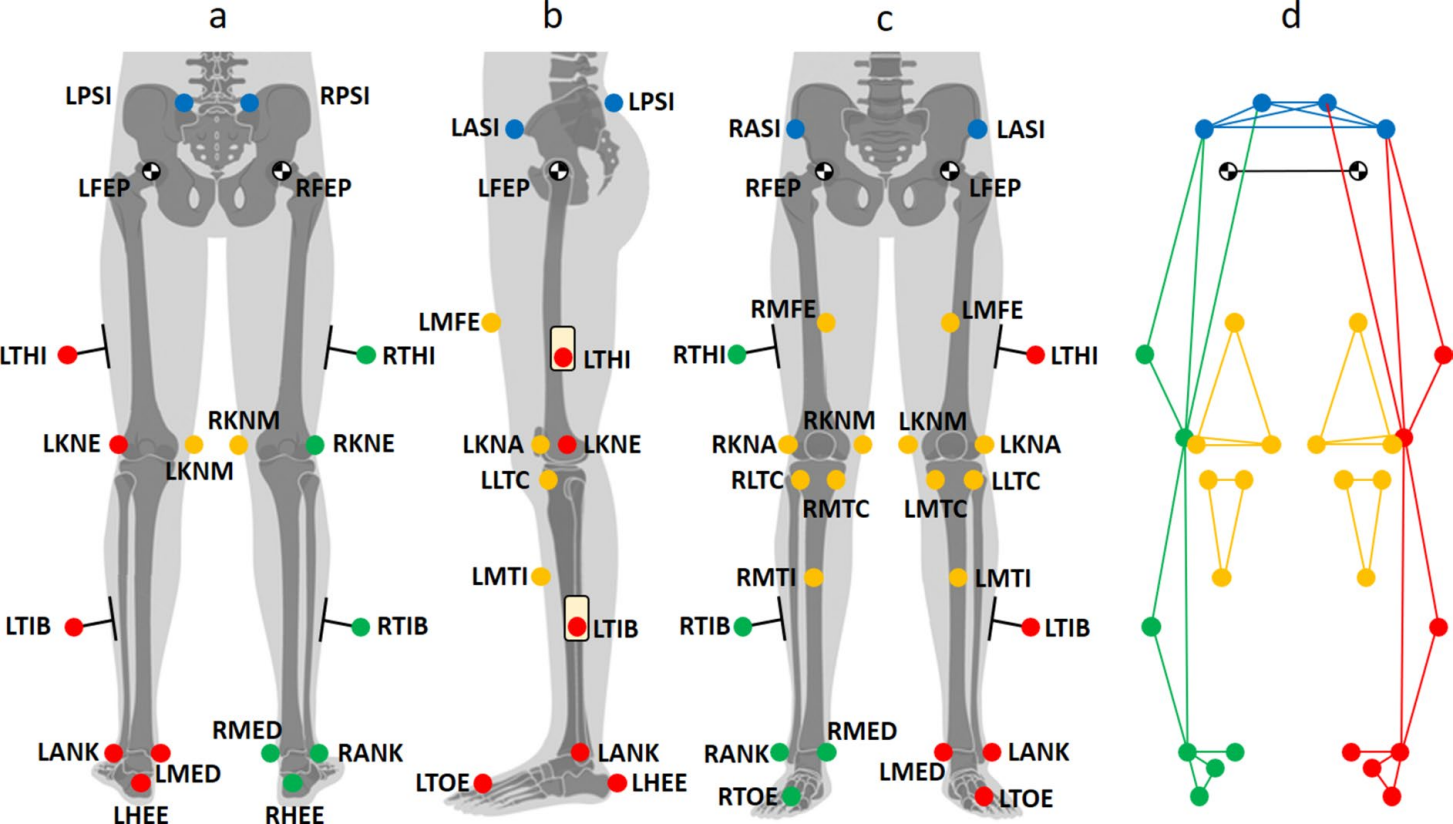
Marker placement in dorsal (**a**), sagittal left (**b**), and frontal (**c**) views; resulting in the complete spatial model (**d**) with 30 markers (Helen-Hayes approach: blue = pelvis, red = left leg, green = right leg; Additional markers according to Kim et al.^23^ are labelled yellow). LFEP and RFEP represent the anthropometrically determined hip joint centers.

The mathematical implementation of the MiKneeSoTA method is based on the extended Helen-Hayes marker model^7^ shown in Fig. 1. Essentially, the post-processing algorithm generates and fits a cylinder into each of the four segments of the lower extremities (left and right thigh and shank). The cylinder is constructed from the static trial of the motion analysis and consists of three reference levels, each containing 12 virtual points. The relative position and orientation of the cylinders with respect to each segment are optimized frame by frame in the dynamic trials to closely resemble their initial position in the static trial. Using the motion of these cylinders, the rotations and translations along the anatomical axes of the lower extremities are calculated.

The present study protocol and all methods used in this study complied with the principles of the Declaration of Helsinki and was approved by the ethics committee of the Hannover Medical School (ethics vote no.: 10861, approval date: 12-04-2023). Prior to participation, written informed consent was obtained from all fifteen healthy volunteers included.

### Measurement protocol

The volunteers were equipped with the necessary Helen-Hayes marker model^7^, extended by six additional markers per limb as required by the MiKneeSoTA method, and then underwent gait analysis while walking on a treadmill. A static trial was captured for each subject prior to the execution of dynamic trials. Gait speed was standardized to 4 km/h and nine consecutive gait cycles were conducted after a familiarization phase of two minutes. Kinematic data was acquired with a motion capture system (200 Hz, six M3 and six M5 Miqus cams each, Qualisys AB, Göteborg, Sweden) and preprocessed using the corresponding Qualisys Track Manager (QTM, Vers. 2023.2). Resulting C3D-Files were finally processed using Nexus (Vers. 1.8.5, Vicon Motion System Ltd., Oxford, UK) for event detection and for the joint angle calculations according to the Helen-Hayes approach. All marker trajectories of the MiKneeSoTA markerset, including the anthropometrically determined hip joint centers from the Helen-Hayes approach (LFEP and RFEP), were exported and optimized with the MiKneeSoTA algorithm.

### Marker model

In accordance with Kim et al.^23^, the inclusion of twelve additional markers (six per limb) employed to the Helen-Hayes approach^7^ (Fig. 1, Table 1), results in an overdetermination of the four limb segments (right shank, left shank, right thigh, and left thigh). These additional markers (yellow) are used to track knee joint landmarks affected by only minimal soft tissue displacement, as well as the positions of the medial intercept of the knee and ankle mediolateral axes (left = red and right = green). Rotational offset determination for the knee joint involved the use of wand markers, which were aligned in the neutral-zero position of the legs. Therefore, volunteers were instructed to stand with their feet at zero degrees of external foot rotation (parallel medial foot edges). The wand markers were aligned in this position using a taut string as a reference to ensure they were in line with the markers (i.e., RTHI between RFEP and RKNE; and RTIB between RKNE and RANK for the right leg) forming a triangle in the frontal plane for each segment along the sagittal plane. This setup assigns an offset to the segments modeled based on these triangles, eliminating the need for subject-specific correction values that would otherwise need to be obtained.

**Table 1:**
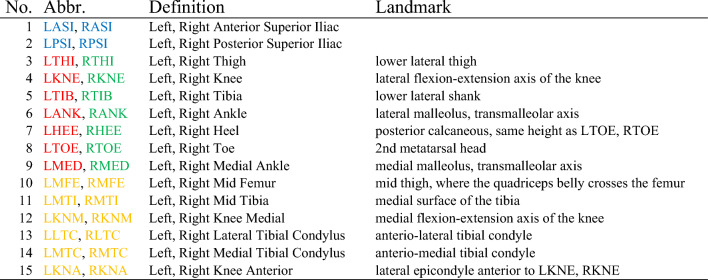
List of markers used in Fig. 1. Markers 1–9 represent the Helen-Hayes approach^7^ with additional medial ankle markers (LMED, RMED); Markers 10–15 represent coronal thigh and shank planes according to Kim et al.^23^.

### Segment modeling

The left and right lower limbs are each modelled as consisting of two segments: the thigh (*t*) and shank (*s*). For each segment, the method calculates the radius of a virtual best fit cylinder. This is achieved by first calculating *three different radii* at three transverse reference levels: bottom (*b*), mid (*m*), and top (*t*). Except for the thigh mid-level, the vector coordinates of two selected markers during a static trial are used to define a centroid position and radius (*r*) for each level. Centroid coordinates (*X*_*b*_, *Y*_*b*_, *Z*_*b*_ for the bottom plane; *X*_*m*_, *Y*_*m*_, *Z*_*m*_ for the mid plane; and *X*_*t*_, *Y*_*t*_, *Z*_*t*_ for the top plane) are given by the coordinates of the global midpoints between the assigned pair of markers (Table 2), with two exceptions. For the thigh top level, *Z*_*t*_ is defined to be at the global Z-coordinate of the hip joint center (RFEP, LFEP). For the thigh mid-level, *Z*_*m*_ is estimated from the mean Z-coordinate of markers 3 and 4 (KNE and THI, Table 1), while the X- and Y-coordinates are estimated from the midpoint between projections of the thigh top and bottom level centroids onto the global transverse plane.

**Table 2:** List of assigned marker pairs exemplarily for the right leg. Corresponding markers for the left leg are analogous.

Segment	Plane	Marker pair
Thigh	Top	RMFE, RFEP
	Mid	–
	Bottom	RKNA, RKNM
Shank	Top	RMTC, RLTC
	Mid	RMTI, RTIB
	Bottom	RANK, RMED

For the right extremity, the radii of the three reference levels for the thigh and shank are calculated as follows (Eq. 1):

1 $$ \begin{aligned}    & r_{{t,t}}  = 0.5\left\| {\overrightarrow {{RMFE}}  - \overrightarrow {{RFEP}} } \right\|\quad\,& r_{{s,t}}  &= 0.5\left\| {\overrightarrow {{RMTC}}  - \overrightarrow {{RLTC}} } \right\| \\   &   r_{{t,m}}  = 0.5(r_{{t,t}}  + r_{{t,b}} )\qquad\qquad\quad\,\,\,\,\,& r_{{s,m}} &= 0.5\left\| {\overrightarrow {{RMTI}}  - \overrightarrow {{RTIB}} } \right\| \\ &    r_{{t,b}}  = 0.5\left\| {\overrightarrow {{RKNA}}  - \overrightarrow {{RKNM}} } \right\|\,\, &r_{{s,b}}  &= 0.5\left\| {\overrightarrow {{RANK}}  - \overrightarrow {{RMED}} } \right\| \\  \end{aligned}  $$

The radii of all three levels of each segment are subsequently averaged to obtain the final radius of each of the corresponding best fit cylinders *(r*_*t,cyl*_ and *r*_*s,cyl*_*)* as follows (Eq. 2):

2 $$ r_{{t,cyl}}  = \frac{{r_{{t,t}}  + r_{{t,m}}  + r_{{t,b}} }}{3}\quad r_{{s,cyl}}  = \frac{{r_{{s,t}}  + r_{{s,m}}  + r_{{s,b}} }}{3} $$

**Fig. 2:**
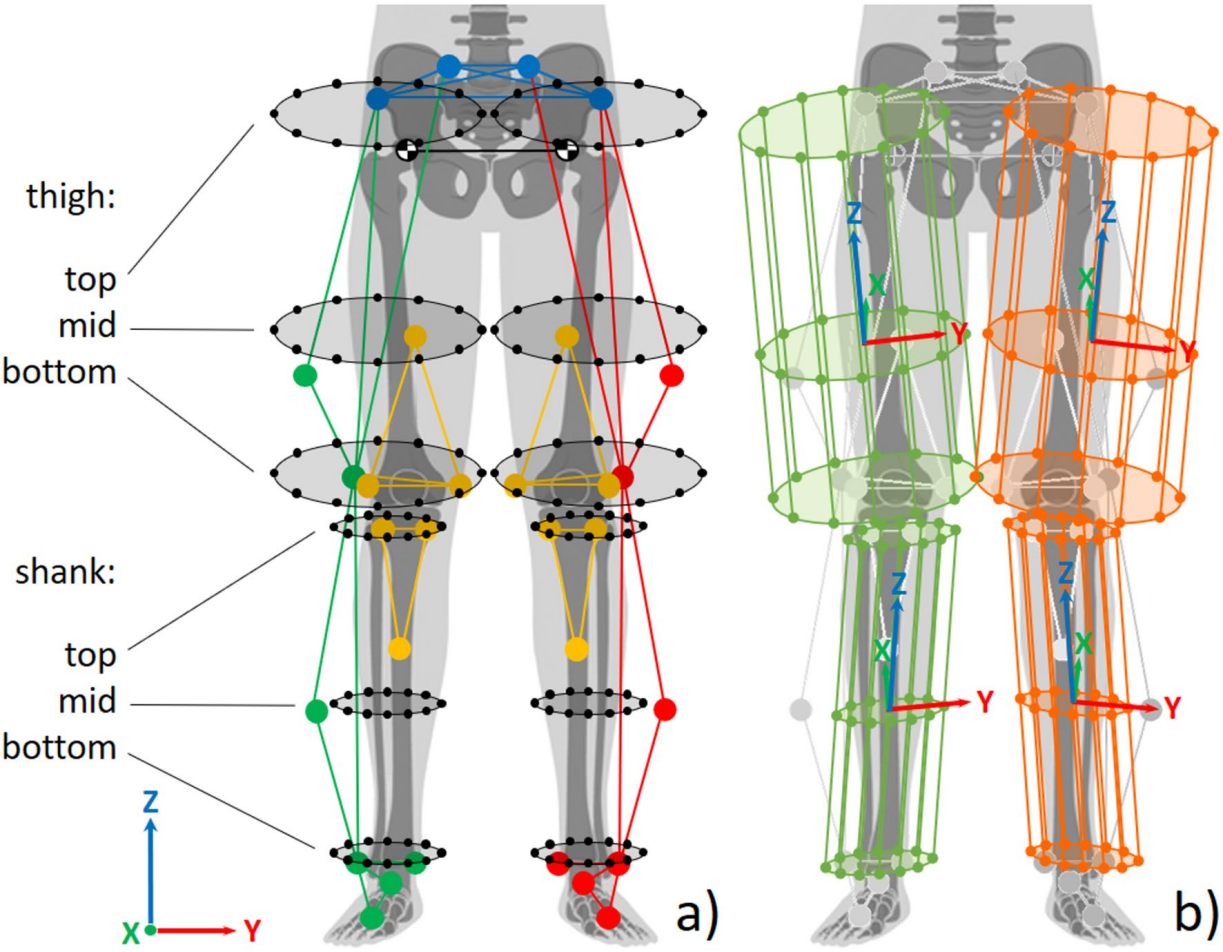
Best fit virtual cylinder generation based on the marker model in Fig. 1d. Taking into account three horizontal reference planes (top, mid, bottom) in the global coordinate system, three circles (grey) consisting of twelve evenly distributed virtual points (black) are generated for each segment (**a**). The resulting cylinders are then aligned to the anatomical coordinate systems (**b**), with the longitudinal axis of each segment being established first. The left leg is shown in orange, and the right leg in green.

Radii for the left lower extremity are calculated in a similar way, using the analogous markers on the left side (i.e. LMFE, LFEP, LKNA, etc.). The best fit cylinder for each segment is then defined as consisting of three horizontal circular cross-sections (bottom, mid, and top; gray layers, Fig. 2a). The center of the cylinder’s bottom cross-section (*cent*_*b*_) lies at the centroid of the bottom reference level (i.e. at *X*_*b*_*, Y*_*b*_*, Z*_*b*_). The X- and Y-coordinates of the centers of the mid and top cylinder cross-sections (*cent*_*m*_ and *cent*_*t*_, respectively) are likewise *X*_*b*_ and *Y*_*b*_ for both cross-sections. The height of the best fit cylinder (and thus Z-coordinate of the center of the cylinder’s top cross-section) is calculated from the Euclidean distance between the centroid of the bottom reference level and the centroid of the top reference level.

For each reference plane, a circle made up of twelve evenly distributed virtual points is generated (black, Fig. 2a). The coordinates for each point *i* on the respective plane *j* are calculated as follows (Eqs. 3, 4):

3 $${p}_{i,j}=\left(\begin{array}{ccc}{X}_{b}& +& {r}_{cyl}\cdot cos\left(i\cdot \frac{360^\circ }{12}\right)\\ {Y}_{b}& +& {r}_{cyl}\cdot sin\left(i\cdot \frac{360^\circ }{12}\right)\\ {Z}_{b}& +& {\Delta }_{b, j}\end{array}\right)$$

4 $$ {\text{where}}\;\Delta _{{b,b}}  = 0,\;\Delta _{{b,m}}  = \left\| {\overrightarrow {{cent_{m} }}  - \overrightarrow {{cent_{b} }} } \right\|\;{\text{and}}\;\Delta _{{b,t}}  = \left\| {\overrightarrow {{cent_{t} }}  - \overrightarrow {{cent_{b} }} } \right\|. $$

Next, the orientation of the cylinder needs to be adjusted to align its longitudinal axis with the anatomical longitudinal axis of the segment. This is achieved by calculating a rotation axis from the cross product of the current cylinder (longitudinal) axis and the longitudinal anatomical axis of the corresponding segment (expressed relative to the global frame). The anatomical axis is determined by the vector pointing from the centroid position of the bottom reference level to the centroid position of the top reference level, as initially obtained from marker coordinates. The angle of rotation is obtained using the standard formula to calculate the angle between the same two vectors. The newly calculated rotation axis and angle of rotation are then used to appropriately transform the cylinder points, resulting in cylinders that are aligned with the anatomical long axis of the corresponding segment (green and orange, Fig. 2b).

### Definition of local coordinate systems of the cylinders

In the next step, the pose of each cylinder’s local coordinate system is calculated relative to the global reference frame, to later be used to determine the relative motion between the segments. The orientation of the cylinder’s local antero-posterior-axis is defined as orthogonal to the longitudinal axis of the cylinder and the global medio-lateral axis. Subsequently, the cylinder’s local medio-lateral axis is determined as the cross product of the cylinder’s local antero-posterior and longitudinal axes. As a result, all cylinder coordinate systems are thus roughly aligned in the same direction. Therefore, the cylinders’ medio-lateral axes of the right leg point medially, whereas those of the left leg point laterally (Fig. 2 and Table 3).

**Table 3:** Directions of coordinate systems used.

Dir	Left leg			Right leg		
	X-axis	Y-axis	Z-axis	X-axis	Y-axis	Z-axis
+	Posterior	Lateral	Proximal	Posterior	Medial	Proximal
−	Anterior	Medial	Distal	Anterior	Lateral	Distal

### Optimization algorithm

**Fig. 3:**
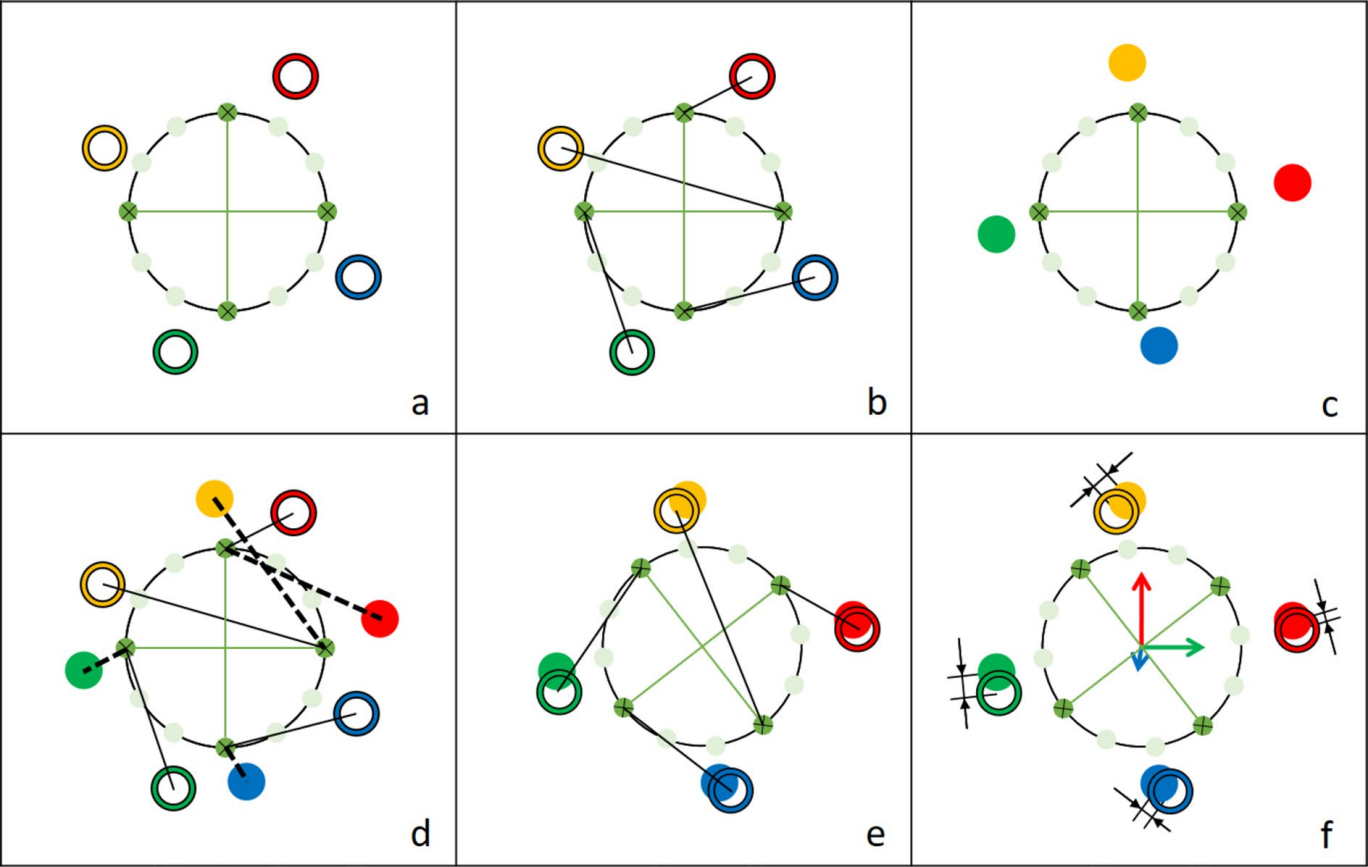
Optimization procedure. (**a**) Based on the marker vectors described in Eq. (1), a best fit cylinder is generated for each segment (of which a single 2D horizontal cross-section is illustrated here) within the static trial; (**b**) Reference distances are calculated between the virtual cylinder points and the real markers (exemplified here by only four connections between virtual cylinder points and real markers); (**c**) In most frames of the dynamic trial, the positions of the real markers naturally differ from their reference positions during the static trial; (**d**) Reference distances to the displaced real markers are calculated (dotted lines); (**e**) The pose of the cylinder is modified to match the reference distances from the static trial; (**f**) This is achieved by minimizing the sum of all distance differences.

The optimization algorithm essentially first considers the static trial (Fig. 3a) to calculate reference distances between each optical marker of a segment and the virtual cylinder cross-section points (Fig. 3b). During the dynamic trials (Fig. 3c), the positions and orientations of the underlying reference cylinders, described by six parameters for the 6DOF of the tibiofemoral joint (three rotations and three translations), are optimized by considering the current distances between the markers and the cylinder reference points (Fig. 3d). The poses of the reference cylinders are then modified (Fig. 3e), such that the difference in the sum of these distances between the dynamic and static trials is minimized (Fig. 3f). For computational efficiency, the transformation of the cylinders is performed using homogeneous transformation matrices. This allows the virtual cylinder points to be computed with a single matrix–vector product rather than a sum of two matrix–vector products.

After convergence through optimization, the 6DOF values corresponding to the optimal solution for each frame are saved. Once all frames have been optimized, all three knee joint rotations and translations are calculated based on the corresponding pairs of thigh and shank coordinate systems. Joint angles are expressed relative to the femoral frame and calculated as the YXZ intrinsic rotation sequence (axis directions in Fig. 2, right) that transforms the femoral frame into the tibial frame, comparable to Grood and Suntay’s joint coordinate system^24^. For convenience, flexion, adduction and external rotation are expressed as positive rotations. Finally, kinematic profiles were smoothed using the cubic ‘smooth.spline’ function in the R statistics package (Version 3.6.2)^25^. Importantly, however, smoothing splines in this context were used strictly to reduce the presence of random noise in otherwise unfiltered data (maximum smoothing parameter of 0.4, in addition to visual inspection to rule out overfitting).

To compare MiKneeSoTA-based kinematics against Helen-Hayes estimates, the mean joint rotations as well as the corresponding standard deviations were calculated across all nine trials of each subject. For additional comparison against previously published experimental data, the selected datasets and the average kinematics of all trials and subjects gained with the MiKneeSoTA method were adapted to follow the same sign conventions, as well as joint angle and joint translation representations (specifically, clinical translations *q*), as those described by Grood and Suntay^24^. Similarly, the external data was post-processed to optimise reference frame orientations^26^, thus ensuring visible differences between the curves could not possibly be attributed to differences in joint coordinate definitions. An adaption of this method was also performed for the translational data.

## Results

### Comparison of methods

**Fig. 4:**
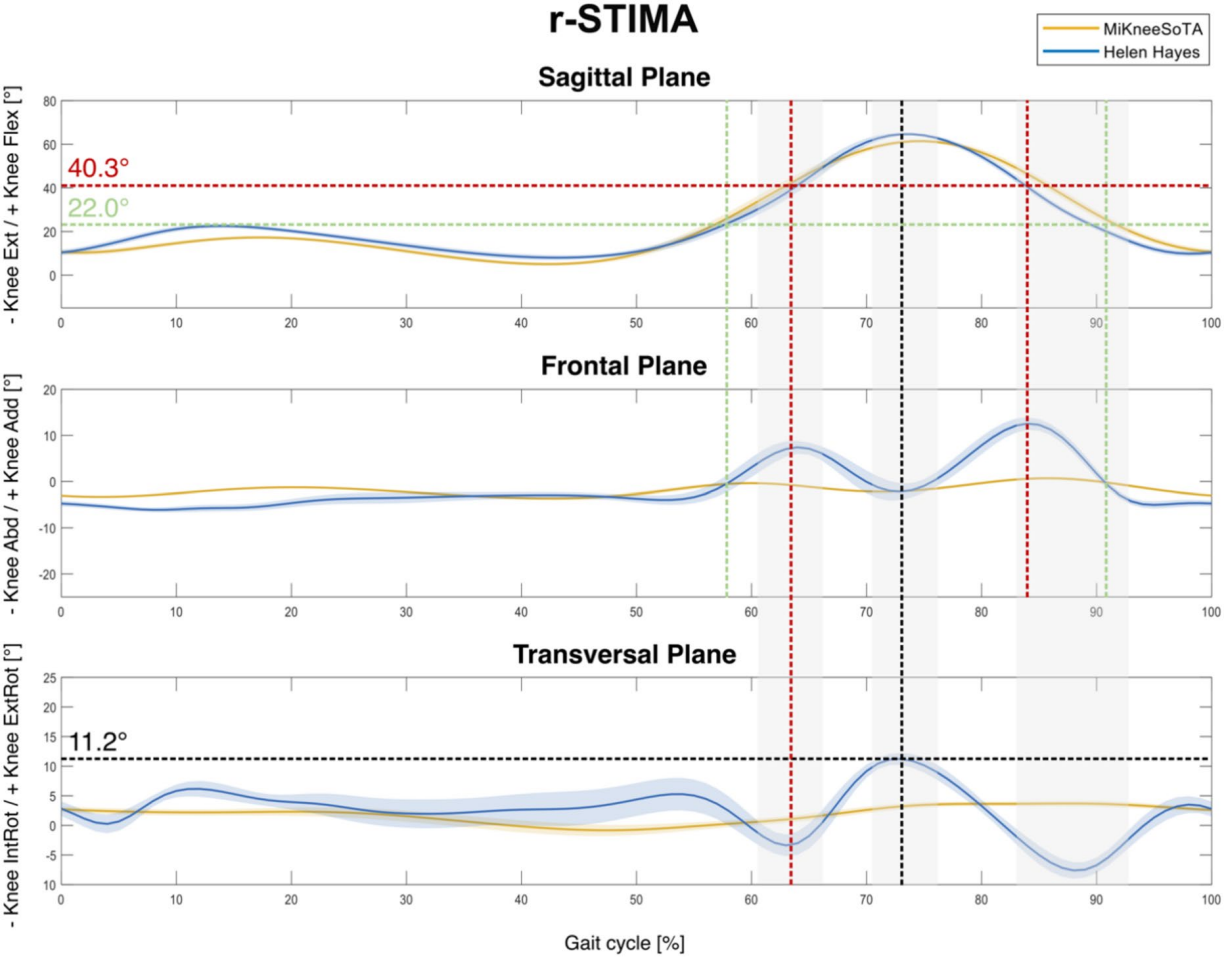
3D knee kinematics (mean ± SD from nine steps) comparing MiKneeSoTA (yellow) and Helen-Hayes approach (blue) for r-STIMA. Green dashed lines indicate start/end of frontal plane deviation of the Helen-Hayes approach and the corresponding values in the sagittal plane (knee flexion) at those timepoints. The red dashed lines indicate the point of maximum deviations in the frontal plane. Grey shaded areas highlight main intervals of transversal plane deviations and the corresponding intervals for the other two planes. Black dashed lines denote maximum knee external rotation, corresponding with the highest knee flexion in the Helen-Hayes approach.

The resulting knee kinematics from the MiKneeSoTA method (yellow) and the standard Helen-Hayes approach (blue) are compared for each individual subject and displayed here for three sample subjects (Fig. 4). In the interpretation, the first subject clearly showed raised STIMA (r-STIMA) using the Helen-Hayes approach (Fig. 4); the second subject showed moderate STIMA (m-STIMA) using the Helen-Hayes approach (Supplementary Material); and the third subject only scarcely showed STIMA (s-STIMA; Supplementary Material).

In the sagittal plane, knee kinematics of the r-STIMA subject obtained by both methods show comparable angular profiles. This is not the case for the frontal and transverse planes, where they noticeably differ from each other. Compared to the MiKneeSoTA method, the knee kinematics obtained through the Helen-Hayes approach reveal the manifestation of striking profile fluctuations accompanied by higher standard deviations in both of these planes. When considering the timepoints of the onset and conclusion of these deviations (indicated by the green dashed line) and of their local maxima (indicated by the red dashed lines) in both the frontal (knee adduction) and the sagittal plane (Fig. 4), it becomes evident that they occur during similar knee flexion angles in the swing phase. On average, the deviation onset and termination take place at 22.0° and their maxima at 40.3° knee flexion.

Additionally, the first deviation maximum in the frontal plane (occurring during the swing phase) seems to be correlated with the occurrence of the first local minimum of the Helen-Hayes approach in the transverse plane, whereas the second maximum does not. Instead, a distinct temporal correlation becomes evident when knee joint angles in the sagittal plane are taken into consideration. During the intervals where the Helen-Hayes and the MiKneeSoTA method show the highest differences in the sagittal plane (approx. 83–93% of gait cycle), corresponding considerable differences in the transverse plane can be observed (last grey shaded area). In fact, similar effects can be observed earlier in the swing phase as well (first and second grey shaded areas). The increase and decrease of knee flexion angle within the swing phase clearly leads to increased profile fluctuations with the Helen-Hayes approach compared to the MiKneeSoTA approach, whereby the maximum knee flexion exceeds that observed with the MiKneeSoTA method.

The underlying characteristics of the Helen-Hayes method appear to exert a direct impact on the knee joint rotation values measured in the transverse plane. The time-shifted, sharper increase of knee flexion seems to result in increased internal tibial rotation (first grey shaded area). The same applies with extension of the knee during the terminal swing phase (last grey shaded area). In contrast, the increased knee flexion resulting from the Helen-Hayes approach at maximum flexion during the swing phase correlates with substantial external tibial rotation of up to 11.2° (indicated by the black dashed lines).

When looking at the m-STIMA subject (Supplementary Material, Fig. S1), comparable but relatively milder effects in the frontal plane can be observed. The deviation onset takes place at 15.3° (green dashed lines) and its maxima at 41.2° of knee flexion (red dashed lines). The increase and decrease of the knee flexion angle during swing phase captured with the Helen-Hayes approach also shows increased dynamics and a surpassing of the kinematics measured with the MiKneeSoTA method. This can also be observed at the end of the swing phase, when the knee joint once again undergoes increasing extension (second grey shaded area), and a local minimum in transversal knee rotation is observable. As already seen with the r-STIMA subject, maximum external knee rotation correlates in time with the maximum knee flexion for the Helen-Hayes approach.

The s-STIMA subject shows almost comparable joint angle profiles in the sagittal and frontal plane kinematics for both the Helen-Hayes and MiKneeSoTA methods (Supplementary Material, Fig. S2). In fact, the kinematic values from both methods hardly differ from each other in these planes. Despite these similarities, the correlation of kinematic effects between the sagittal and transverse planes (grey shaded intervals) is similar to that seen for the r-STIMA and m-STIMA subjects.

### Comparison with experimental data

The 6DOF knee kinematics derived from all subjects were compared against two distinct experimental datasets extracted from literature^1,8^. Anthropometrics and gait speeds were roughly comparable between the present subjects and the subjects of the literature datasets (Table 4).

**Table 4:** Study characteristics with regard to included subjects.

	MiKneeSoTA	Gray et al.^1^	Lafortune et al.^8^
n	15	15	5
Age (years)	27.5	30.5	27.2
Height (m)	1.765	1.680	1.806
Weight (kg)	70.6	67.4	75.2
Gait speed (km/h)	4.0	4.6	4.3
Study condition	Treadmill	Overground	Overground

**Fig. 5:**
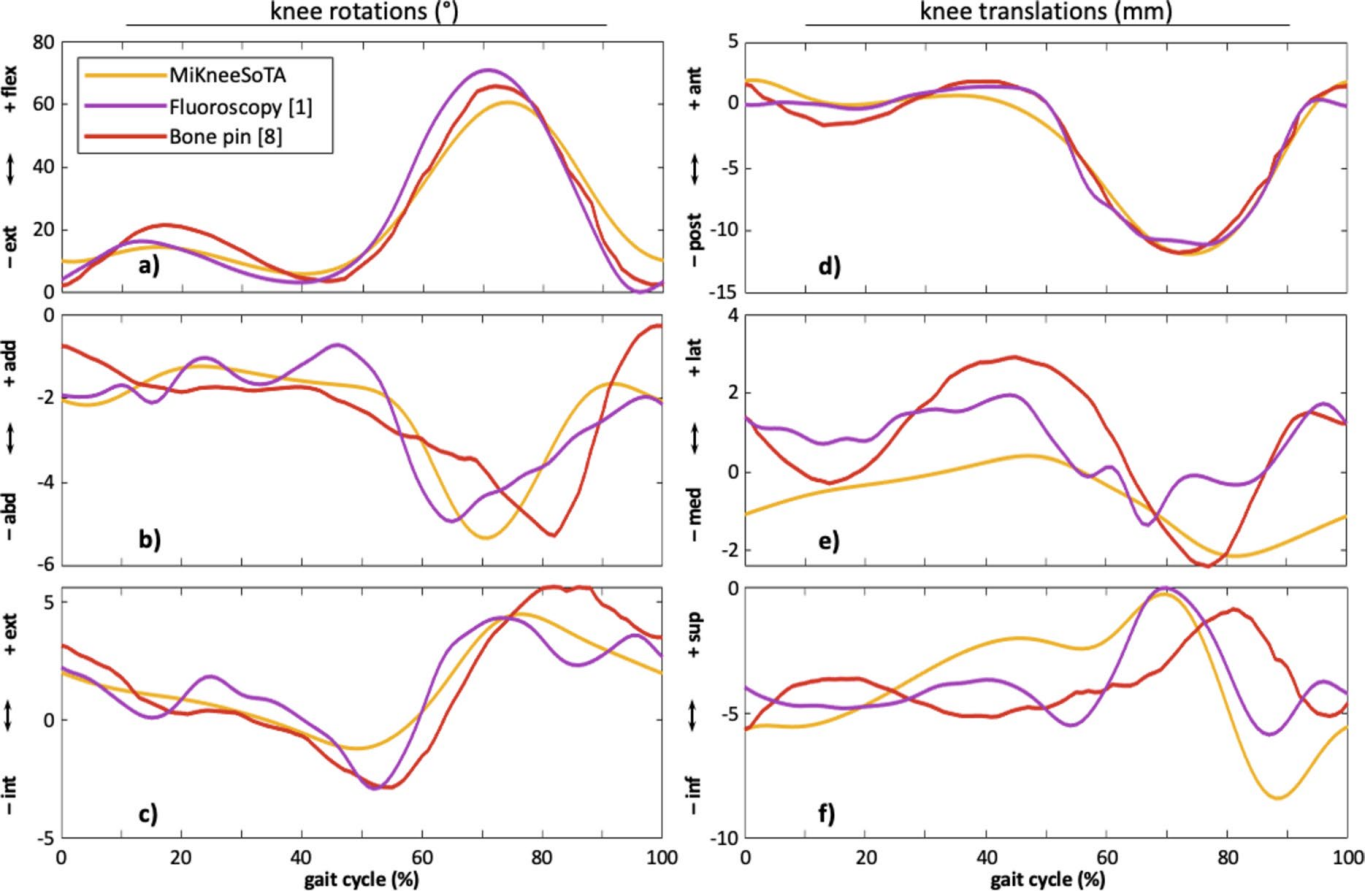
Averaged 3D knee rotations (**a**–**c**) and knee translations (**d**–**f**) resulting from subjects with the MiKneeSoTA method (yellow), with the fluroscopy method^1^ (purple), and with the bone pin method^8^ (red).

Strikingly congruent knee joint angle profiles manifest across all three planes when juxtaposing the kinematics extracted through the MiKneeSoTA method (yellow) against those acquired through the fluoroscopy method by Gray et al.^1^ (purple) and the bone pin method by Lafortune et al.^8^ (red) (Fig. 5a–c). Kinematics from the MiKneeSoTA method show a slightly increased knee flexion angle during the initial and the terminal stage of the gait cycle, with the maximum knee flexion during the swing phase slightly delayed compared to kinematics from both experimental methods (Fig. 5a). In the frontal plane, the joint angle given by the MiKneeSoTA method remains closely aligned but with less fluctuations than with that derived from the experimental fluoroscopy method throughout the stance phase. Nevertheless, a recognizable disparity emerges during the swing phase, where the joint angles reach their maximum abduction at different timepoints in all three datasets. The frontal plane joint angle profile given by the bone pin method shows higher starting values and reaches maximum abduction at the latest (Fig. 5b). In the transverse plane, the joint angle profiles exhibit remarkable homogeneity across all methods, with only marginal differences (Fig. 5c).

The joint translations show a certain degree of concordance between all methods (Fig. 5d–f). There is, for example, a high degree of agreement for the anterio-posterior joint translations (Fig. 5d). However, there are some discrepancies in the medio-lateral translation (Fig. 5e) and the distraction of the knee joint (Fig. 5f). In particular, the range of motion (ROM) measured by MiKneeSoTA in the medio-lateral direction lags behind the other two methods. All three profiles show a tendency of the same movement direction, with the MiKneeSoTA profile being smoother, slightly offset, and subtly time delayed in comparison to the other two profiles. The joint distractions (Fig. 5f) show comparability between the methods, with the MiKneeSoTA method showing the highest ROM, particularly with regard to the swing phase.

## Discussion

### Comparison of methods

As expected, knee kinematics given by the MiKneeSoTA method and the Helen-Hayes approach show just marginal differences with regard to the sagittal plane, which are in addition consistent with the knee flexion/extension angle profiles reported in literature^1,16,18^. Substantial differences only become apparent in the second plane of the joint angle calculations, the frontal plane, which plays a particularly important role when further analyzing the knee joint regarding i.e. the frontal knee joint kinetics^27^. These differences highlight the level of STIMA affecting the Helen-Hayes approach during the swing phase of the gait cycle in particular. Since the onset and termination of the STIMA seem to have an individual character, but seemingly occur consistently at the same knee flexion value in a given subject—i.e. STIMA is triggered in the frontal plane (right before toe-off) at the same flexion angle at which it then abates (during swing extension)—the present results suggest a direct temporal correlation. The raised or moderate STIMA always starts at knee flexion angles between 15° and 25°, whereas maximum STIMA tends to occur at around 40° of knee flexion. We hypothesize this phenomenon to be directly associated with the movement of the iliotibial band upon activation of the tensor fasciae latae muscle, whose trajectory lies directly above the lateral epicondyle of the femur. The lateral knee markers, LKNE and RKNE, are always strategically positioned on this epicondyle during gait analyses. As the knee joint undergoes flexion or extension, this conspicuous tendon dynamically traverses the lateral femoral condyle in the antero-posterior direction. This induces a displacement of the knee marker, which is clearly visible to the naked eye, and was visually documented for the r-STIMA subject using video footage (Supplemental Video material). Relative to the other model markers, this action induces a shift of the lateral knee marker position, contributing to the measurement of a likely artefactual medio-lateral (and antero-posterior) displacement. The extent of this STIMA-driven displacement appears to vary between subjects, likely a reflection of differences in the structural attributes of the tendon. As the marker shifts because of the tendon motion, the effect in the frontal plane is rather pronounced. Therefore, it impacts the knee adduction angle particularly during the swing phase, rendering it susceptible to significant error.

Examining the literature on motion analysis studies of the knee joint reveals that other authors also show comparable anomalies in the form of undulating knee angle profiles in the frontal plane during the swing phase, accompanied by occasionally substantial standard deviations, akin to the straightforward Helen Hayes approach^16,28–32^. For instance, Clemént et al.^30^ and Mezghani et al.^31^ observed kinematic patterns in the frontal plane within their study populations, suggesting that healthy subjects could be classified into different phenotypes. This categorization was primarily attributable to the different kinematic profiles during the swing phase. The differences in motion profiles could on the one hand be indicative of individually pronounced STIMA or, on the other hand caused by differences in local reference frame orientations^26^. In fact, a combination of both is likely to be assumed to explain a considerable portion of the observed subject differences. Nevertheless, the interpretation of phenotypes identified from differences in kinematic data must be drawn very carefully and should always first rule out the possibility that differences are caused by STIMA or reference frame inconsistencies, rather than physically different joint motion patterns. Accordingly, Mahaffey et al.^29^ also rightly caution that their data may be influenced by STIMA in the frontal plane, limiting the generalizability of their findings.

The motion of the Musculus Tensor Fasciae Latae appears to have no direct temporal impact on internal and external knee rotation. Nevertheless, STIMA effects cannot be ruled out in this context for the Helen-Hayes approach, as results show marked fluctuations in the joint angle profiles in this plane. The temporal correlation between external rotation peaks and maximum knee flexion are strongly indicative of STIMA. The increase in knee flexion leads to an augmented displacement of the skin relative to the lateral femoral condyle as previously described. Consequently, the two lateral knee joint markers shift posteriorly relative to the other segment markers, exerting a direct influence on knee joint kinematics in the transverse plane. The present data from the Helen-Hayes approach exhibits varying baseline values for the individual examples (r-STIMA, m-STIMA, and s-STIMA). Therefore, it is likely that the idealized compromise center of knee rotation^33^ does not correspond to the actual knee axis (i.e. the instantaneous screw axis) in this data. This so-called cross-talk effect is frequently mentioned in the literature, prompting numerous attempts to mitigate it through knee alignment devices, determination of the functional knee axis, or post-processing algorithms^26,34–36^. Kadaba et al.^7^ already demonstrated in the 1990s that varying the location of the knee joint axis through lateral knee marker positioning results in certain displacements in the transverse plane. Nevertheless, the MiKneeSoTA method appears to substantially reduce this effect, as the baseline values of knee rotation in all three presented examples are comparable in the transverse plane.

### Comparison with experimental data

The only reliable datasets available for a comparison of knee joint kinematics for the present purposes are derived from LaFortune et al.^8^ and Gray et al.^1^. To the authors’ best knowledge, these are the only two research groups that have comprehensively measured and published the dynamic 6DOF kinematics of the knee joint in healthy subjects during walking in the absence of STIMA. Both applied different methods, unfortunately not readily applicable for everyday use in clinical studies due to ethical considerations. To ensure consistency across all datasets, frame orientations and position were optimized^26^. The results showed that only minor differences in joint motion were present, and it can be confidently asserted that dynamic joint rotations and translations exhibit comparable trends and encouraging levels of agreement.

All three methods yield almost identical kinematic curve patterns, especially regarding the rotational results, with discrepancies primarily arising due to a slightly increased knee flexion in the MiKneeSoTA method and temporal differences in reaching peak knee abduction. These differences can be grounded in the different walking situations used in the studies: all subjects in the two investigated studies walked overground while in our present study the subjects walked on a treadmill. This decision was made to ensure a standardized walking speed for all subjects and thus minimize the kinematic variance in-between different subjects as well as within single subjects. The use of different event detection algorithms to define single gait cycles in the different studies likely also contributed to slightly time shifted profiles.

The fact that the results obtained through the presented novel MiKneeSoTA method match the results by Gray et al.^1^ and Lafortune et al.^8^, whose acquisition methods are free of STIMA, strongly implies that our method can reduce the influence of STIMA in 3D gait analysis. While translational movements in the anterior–posterior direction are almost identical between methods, greater differences are recognizable in the other two translational directions. Since the medio-lateral knee joint shift shows a comparatively small ROM of only a few millimeters, the very different kinematic capturing methods, with additionally different types of measurement errors, must be taken into consideration. In contrast, with a larger ROM, such as in the knee joint distraction (Fig. 5f), the studies tend to be comparably consistent again. Overall, translations captured by the MiKneeSoTA method behave much smoother, especially in the medio-lateral direction and in the joint distraction. In these directions, the bone pin method generally exhibits larger ranges and the fluoroscopy method shows comparatively fluctuating profiles. This fluctuation for the fluoroscopy method is also evident for joint rotations in the frontal and transverse planes. We assume that some kind of a systematic error may have been introduced into the curve formation, perhaps induced by the image processing. Although we have high confidence in that data, it is advisable to critically evaluate these results obtained through fluoroscopy. In addition, we assume that such fluctuating medio-lateral translations can only occur in more pronounced knee instabilities. A healthy knee joint, well-guided by intact ligament and cartilage structures, is unlikely to exhibit such large fluctuations, especially in the medio-lateral direction. Both, the bone pin method and the MiKneeSoTA method, present much smoother and more comprehensible joint translations. Furthermore, we assume that the bone pin method is also subject to systematic errors, as the pins can be moved or bent by the soft tissue impingement. These effects are expected to be smaller than the direct STIMA but could explain the larger ranges observed by Lafortune et al.^8^.

It should be noted that the data comes from different subjects, so differences between the joint angle curves can be expected. Slight differences in gait speed may also alter the angle profile and must be considered. As bone pins inserted to the body may cause pain during walking, altered gait behavior can also not be ruled out in the dataset of Lafortune et al.^8^.

Given the extreme sensitivity, for example, of abduction/adduction and internal/external rotation values (as well as of all three joint translations), to joint orientation and position, we caution against strictly interpreting the presented joint values in degrees or millimeters. Instead, we recommend focusing on the presence and behavior of differences between profiles after accounting for frame effects. From a clinical perspective, adduction during the stance phase of gait would be expected. However, the profile is negative with MiKneeSoTA; this does not necessarily imply mathematical inaccuracy in the data. Upon examining individual gait curves, we observed that female participants primarily exhibited abduction during stance, whereas male participants showed adduction. Overall, more subjects exhibited abduction during stance, resulting in a negative mean profile.

Although the accuracy and precision of the placement of markers on the corresponding landmarks can improve with dedicated training and practical experience, marker positioning will never be perfectly identical from subject to subject, session to session, or observer to observer. Therefore, when working with marker-based systems, kinematic data should be post-processed not only to ensure reproducible and consistent reference frame poses^26^, but importantly also to reduce STIMA effects by using methods like MiKneeSoTA.

## Conclusion

To meet the demand for a motion capture method enabling precise measurement of complete knee joint kinematics, while simultaneously avoiding STIMA and maintaining a non-invasive nature, we introduced the MiKneeSoTA method. By employing this newly developed method, which yields knee joint angle and translation profiles remarkably similar to those in previous experimental studies without STIMA, we are highly confident in having achieved this objective. We appreciate that conclusive evidence that MiKneeSoTA successfully eliminates STIMA in established marker-based optoelectronic gait analysis protocols will require the simultaneous collection of a biplanar fluoroscopy-based ground truth. Nevertheless, our results are strong preliminary evidence that MiKneeSoTA does hold the potential to tackle the challenge of STIMA, especially when juxtaposed with documented observations and understanding of the relevant physiological structure (e.g. tensor fasciae latae) behaviour, and previously published experimental findings. This contrasts with the conventional Helen-Hayes approach, in which systematically incorrect marker movements e.g. caused by the musculus tensor fasciae latae tendon during knee flexion causes STIMA in the swing phase. Notably, STIMA occurs during the swing phase, which cannot be eliminated with the conventional approach. The presented MiKneeSoTA method therefore represents a breakthrough, providing an unbiased and highly precise approach to capture 6 DOF knee kinematics for the first time, unmarred by the presence of soft tissue artefacts.

## Supplementary Information

Supplementary Information 1.

Supplementary Information 2.

Supplementary Video 1.

## Data Availability

The datasets generated and analyzed during the current study are available from the corresponding author on reasonable request.
