# Supplementary material for: A new method called MiKneeSoTA to minimize knee soft-tissue artifacts in kinematic analysis
Source: Sci Rep. 2024 Sep 5;14:20666. doi: 10.1038/s41598-024-71409-z (PMC11377703; doi:10.1038/s41598-024-71409-z)
Supplement: Supplementary file 1 — Supplementary Information 1. [file 41598_2024_71409_MOESM1_ESM.docx]

Additional information

The supplementary material includes two images of subjects classified as moderate STIMA (m-STIMA) and scarce STIMA (s-STIMA), and a video illustrating the knee marker displacement during knee flexion of the subject classified as raised STIMA (r-STIMA). The video shows two knee flexion-extension cycles, with a brief pause in the second sequence coinciding with the critical time points showing the most pronounced STIMA effects, which occur at 22° and 40° of knee flexion in Figure 4.
