# Supplementary material for: A new method called MiKneeSoTA to minimize knee soft-tissue artifacts in kinematic analysis
Source: Sci Rep. 2024 Sep 5;14:20666. doi: 10.1038/s41598-024-71409-z (PMC11377703; doi:10.1038/s41598-024-71409-z)
Supplement: Supplementary file 2 — Supplementary Information 2. [file 41598_2024_71409_MOESM2_ESM.pdf]

A New Method Called MiKneeSoTA to

Minimize Knee Soft-Tissue Artifacts in Kinematic Analysis.

Ann-Kathrin Einfeldt, Leon Budde, Ariana Ortigas-Vásquez,

Adrian Sauer, Michael Utz, Eike Jakubowitz

Supplementary Information file

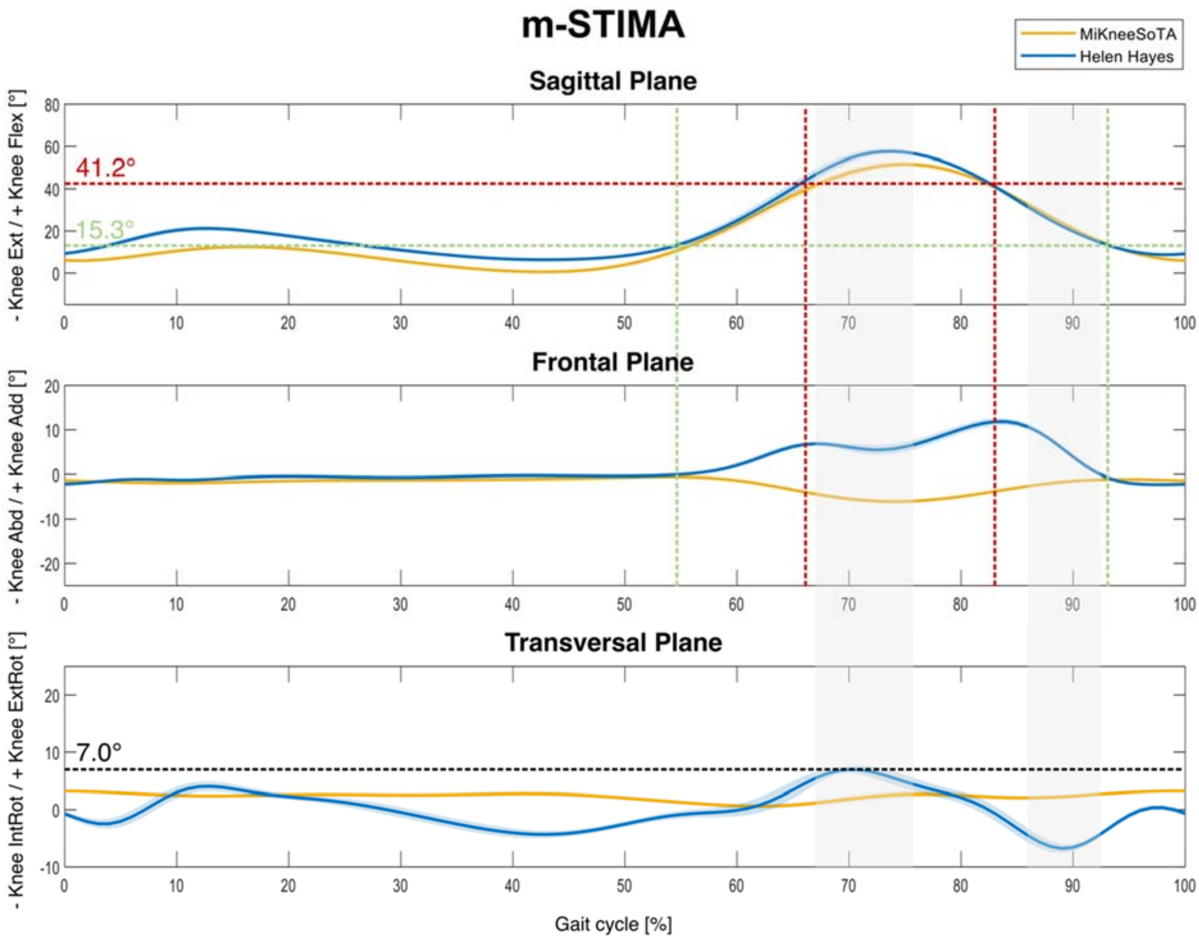

Figure S1: Knee kinematics (mean ± standard deviation out of nine consecutive steps) from the MiKneeSoTA (yellow) and the Helen-Hayes approach (blue) of m-STIMA in all three planes. Green dashed horizontal line marks the amount of knee flexion, where deviation between the two methods in frontal plane starts and ends (green dashed vertical line). Red dashed horizontal line marks the amount of knee flexion, where deviation between the two methods in frontal plane reaches peaks (red dashed vertical line). Grey shaded areas mark the time points where the greatest differences between the two methods in sagittal and transversal plane appear. Black dashed lines mark peak knee external rotation.

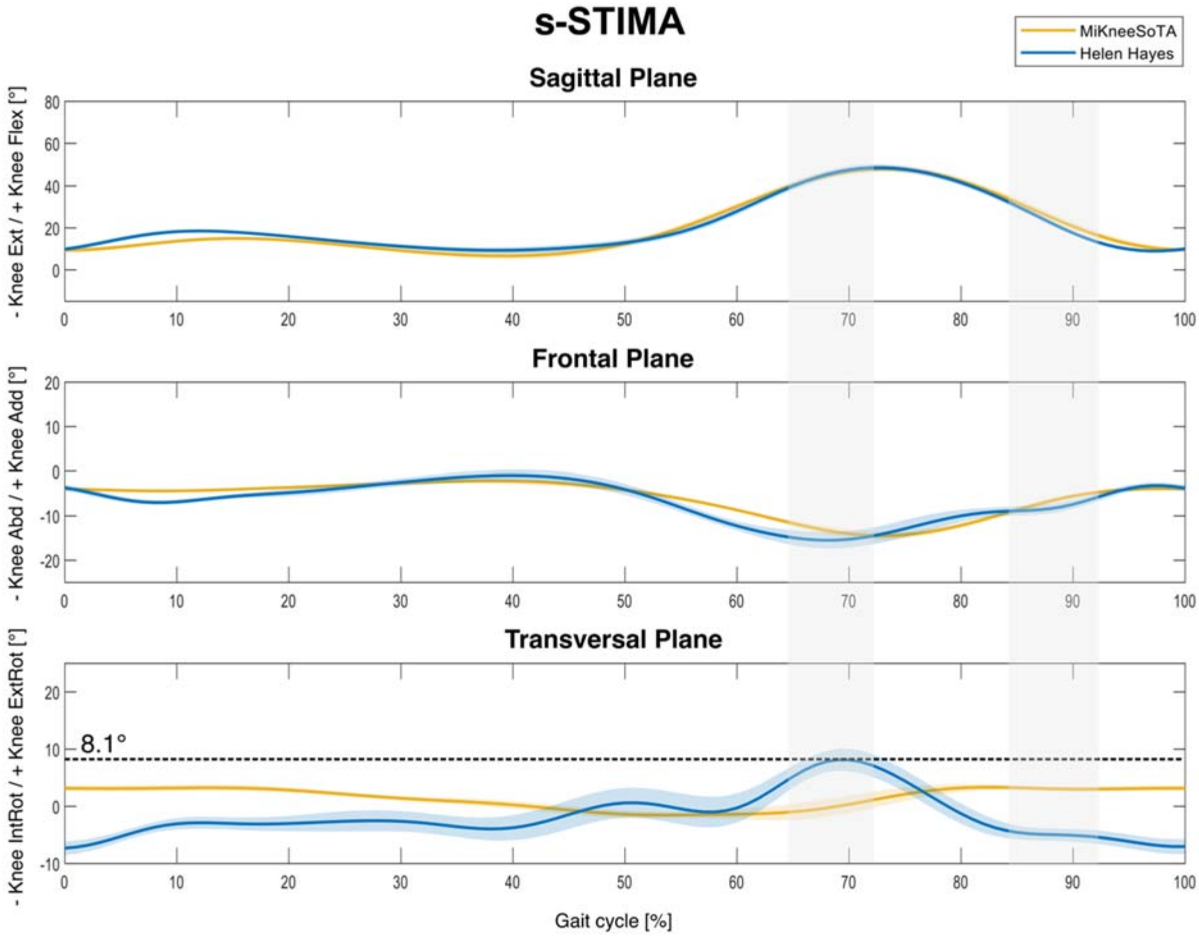

Figure S2: Knee kinematics (mean ± standard deviation out of nine consecutive steps) from the MiKneeSoTA (yellow) and the Helen-Hayes approach (blue) of s-STIMA in all three planes. Grey shaded areas mark the time points where the greatest differences between the two methods in sagittal and transversal plane appear. Black dashed lines mark peak knee external rotation.
